# Supplementary figures and images for: Secondary bile acid ursodeoxycholic acid alters weight, the gut microbiota, and the bile acid pool in conventional mice
Source: PLoS One. 2021 Feb 18;16(2):e0246161. doi: 10.1371/journal.pone.0246161 (PMC7891722; doi:10.1371/journal.pone.0246161)

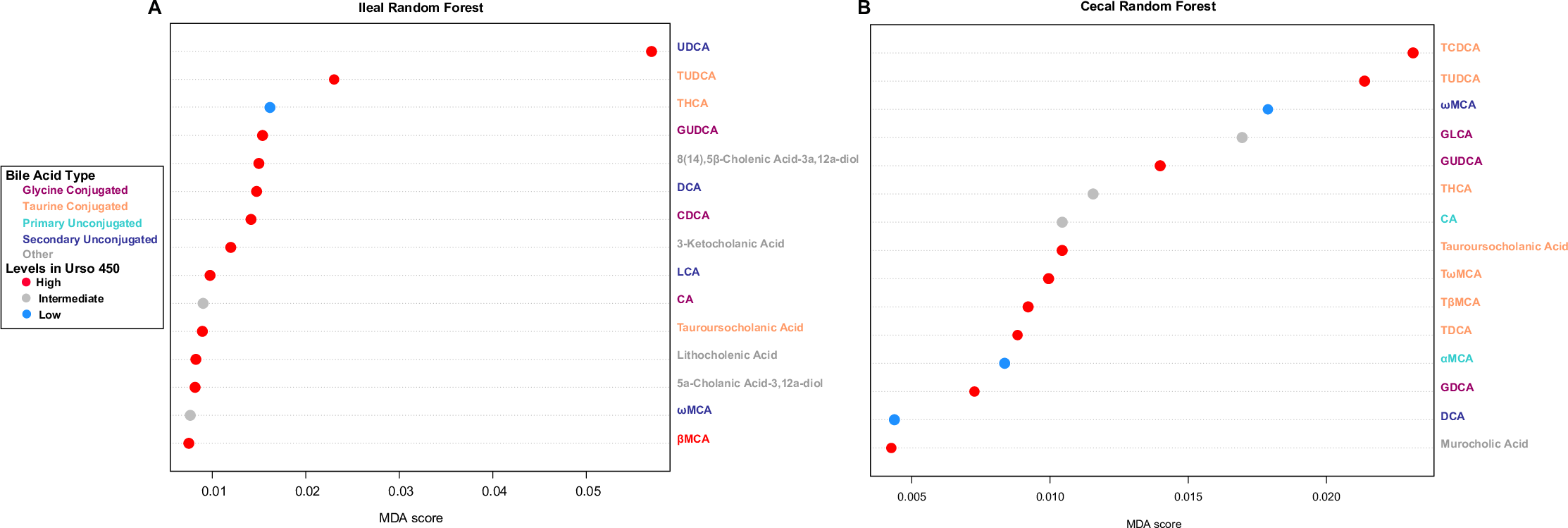

Supplement: S1 Fig — (A) Ileal and (B) cecal principal coordinate analysis (PCoA) biplot using a Spearman correlation for top 10 significant OTUs. (C) Longitudinal fecal principal coordinate analysis (PCoA) biplot using a Spearman correlation for top 10 significant OTUs. (TIF) [file pone.0246161.s001.tif]

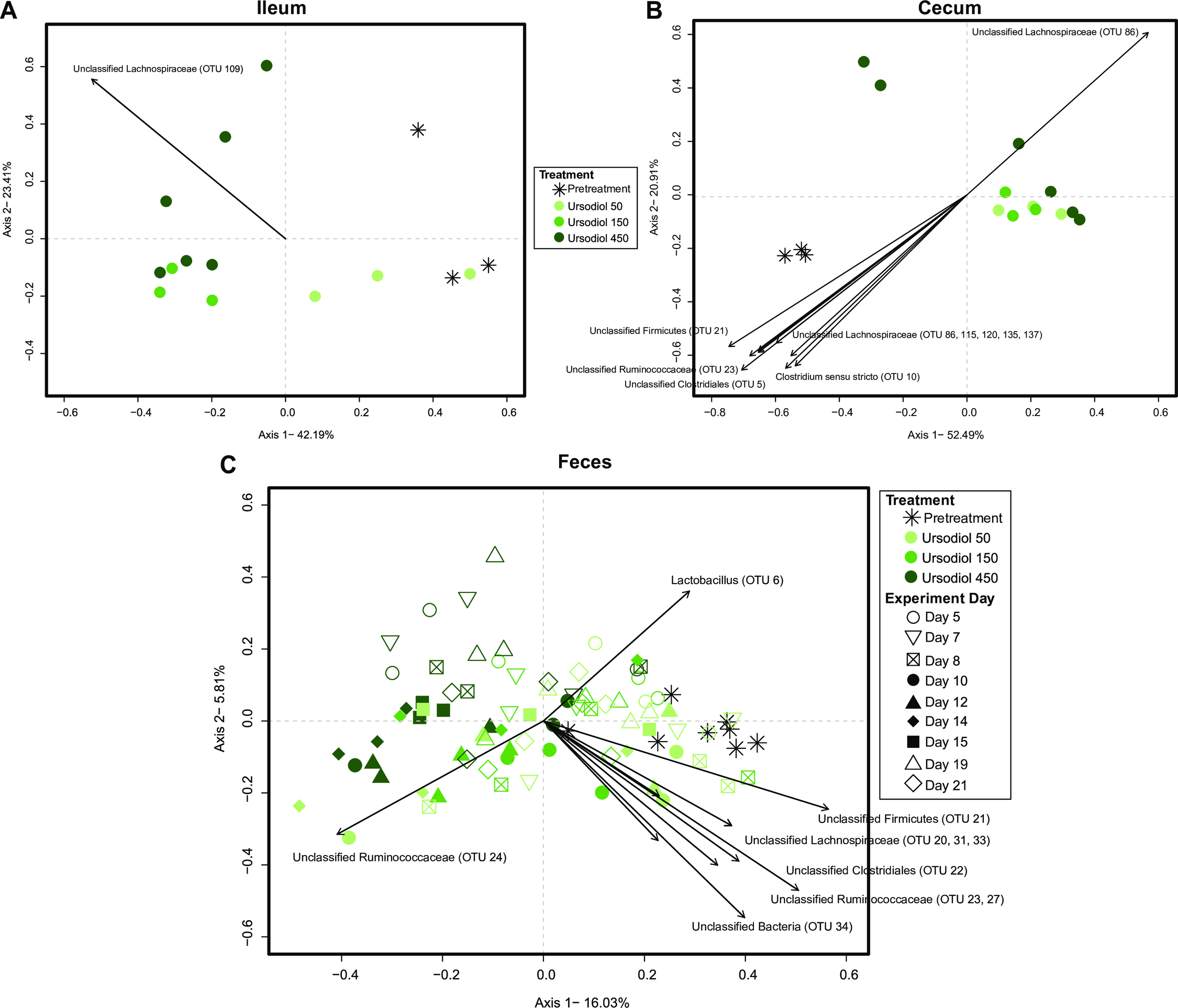

Supplement: S2 Fig — Variable-importance plot of the top 15 bile acids identified by Random Forest analysis in the (A) ileum and (B) cecum. The mean accuracy value decrease (MDA score) is a measure of how much predictive power is lost if the given bile acid is removed or permuted in the Random Forest algorithm. Therefore, the more important a bile acid is to classifying samples into a treatment group, the further to the right the point is on the graph. Bile acid points are color-coded for relative concentrations of each bile acid within the ursodiol 450 treatment group (red if their concentration is high in ursodiol 450 treatment, gray if they were intermediate, and light blue if the concentrations were low). Each bile acid name is colored coded based on bile acid type (purple indicates glycine conjugated, orange indicates taurine conjugated, teal indicates primary unconjugated, blue indicates secondary unconjugated, and gray indicates other type of bile acid). (TIF) [file pone.0246161.s002.tif]

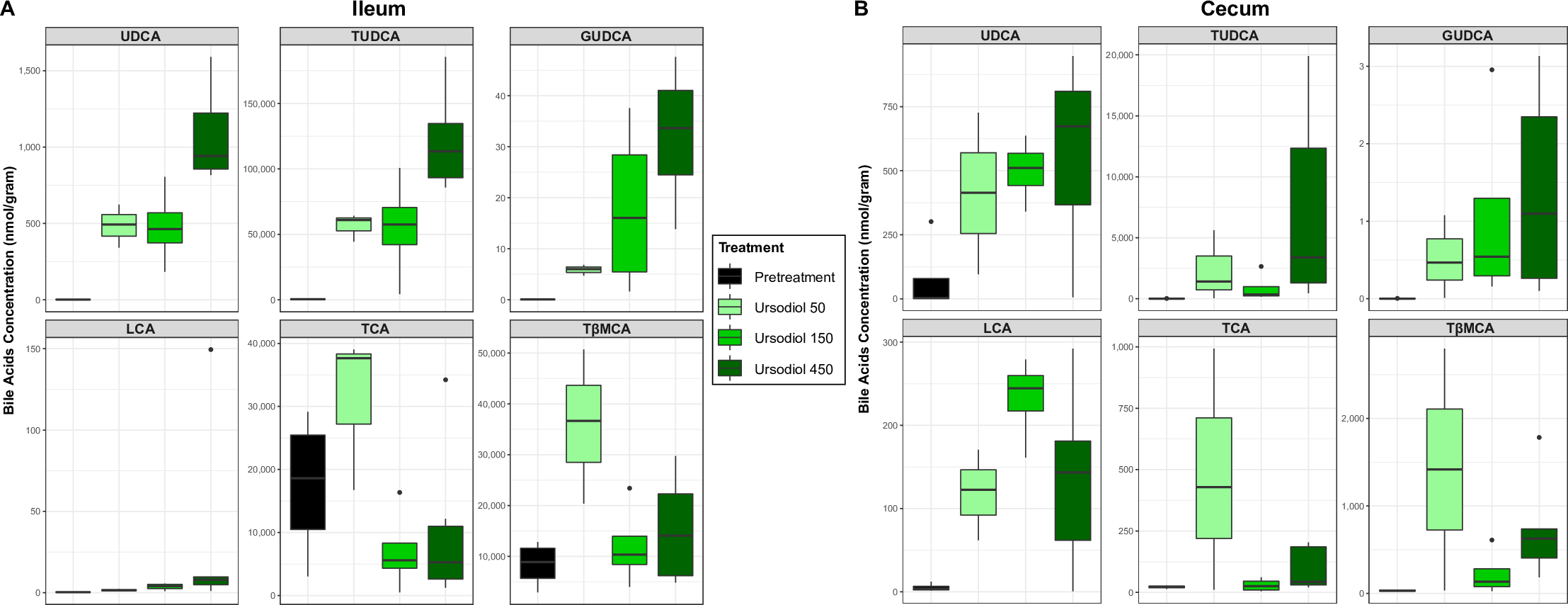

Supplement: S3 Fig — Box and whisker plots of (A) ileal and (B) cecal bile acids that were significantly altered in ursodiol treated mice compared to pretreatment in any of the sample types evaluated (based on a Two-way ANOVA with Dunnett’s multiple comparisons post hoc test). Data represents two independent experiments (pretreatment, n = 4; ursodiol 50, n = 3; ursodiol 150, n = 4; ursodiol 450, n = 6). (TIF) [file pone.0246161.s003.tif]

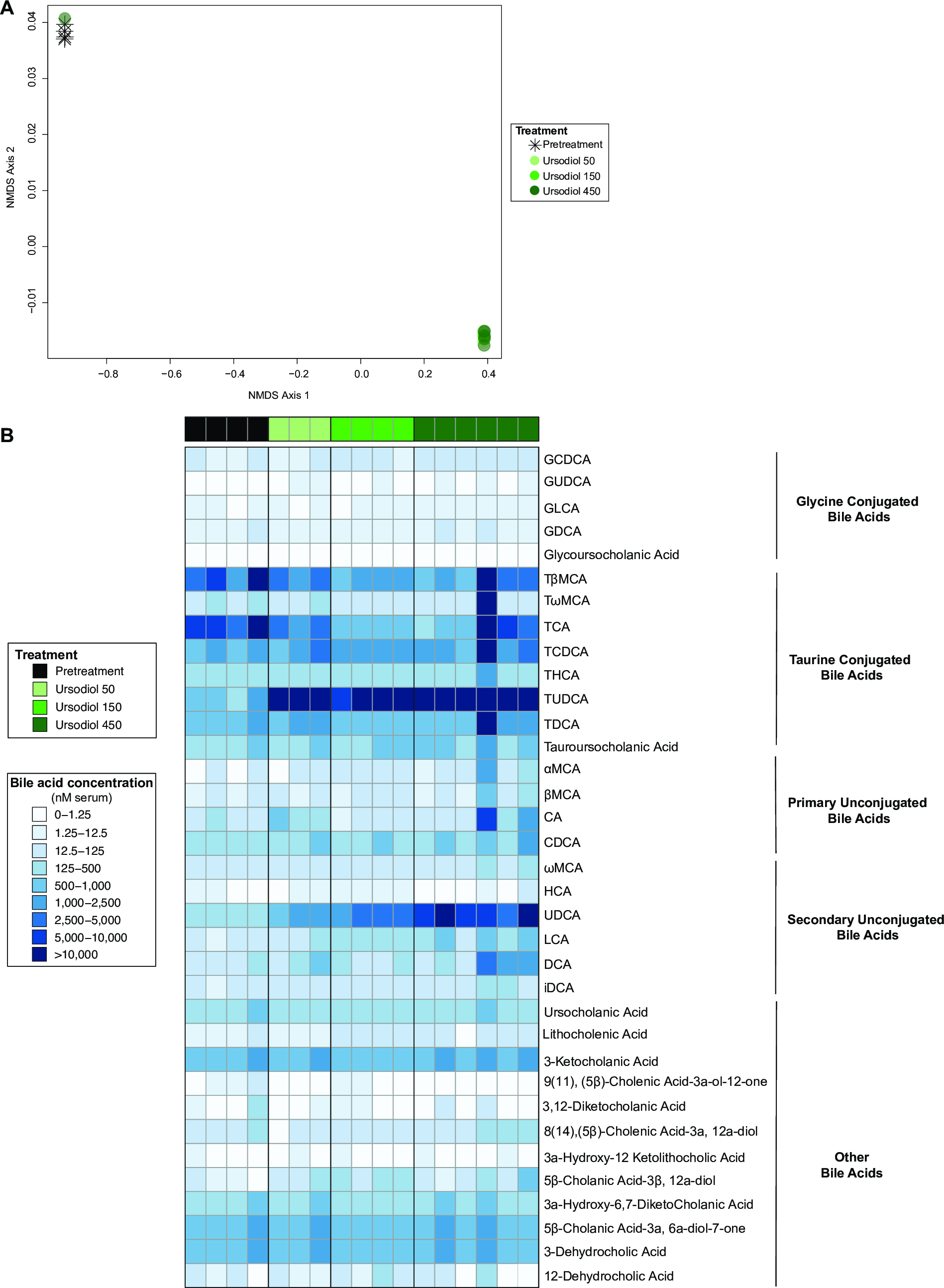

Supplement: S4 Fig — (A) NMDS ordination illustrates dissimilarity indices via Horn distances between bile acid profiles of serum samples. (B) Targeted bile acid metabolomics of murine serum was performed by UPLC-MS/MS and identified 38 distinct bile acids. (C) Variable-importance plot of the top 15 bile acids identified by Random Forest analysis. Bile acid points are color-coded for relative concentrations of each bile acid within the ursodiol 450 treatment group (red if their concentration is high in ursodiol 450 treatment, gray if they were intermediate, and light blue if the concentrations were low). Each bile acid name is colored coded based on bile acid type (purple indicates glycine conjugated, orange indicates taurine conjugated, teal indicates primary unconjugated, blue indicates secondary unconjugated, and gray indicates other type of bile acid). (D) Box and whisker plots of bile acids that were significantly altered in ursodiol treated mice compared to pretreatment in any of the sample types evaluated (based on a Two-way ANOVA with Dunnett’s multiple comparisons post hoc test). Data represents two independent experiments (pretreatment, n = 4; ursodiol 50, n = 3; ursodiol 150, n = 4; ursodiol 450, n = 6). (ZIP) [file pone.0246161.s004.zip › S4_Fig(1).tif]

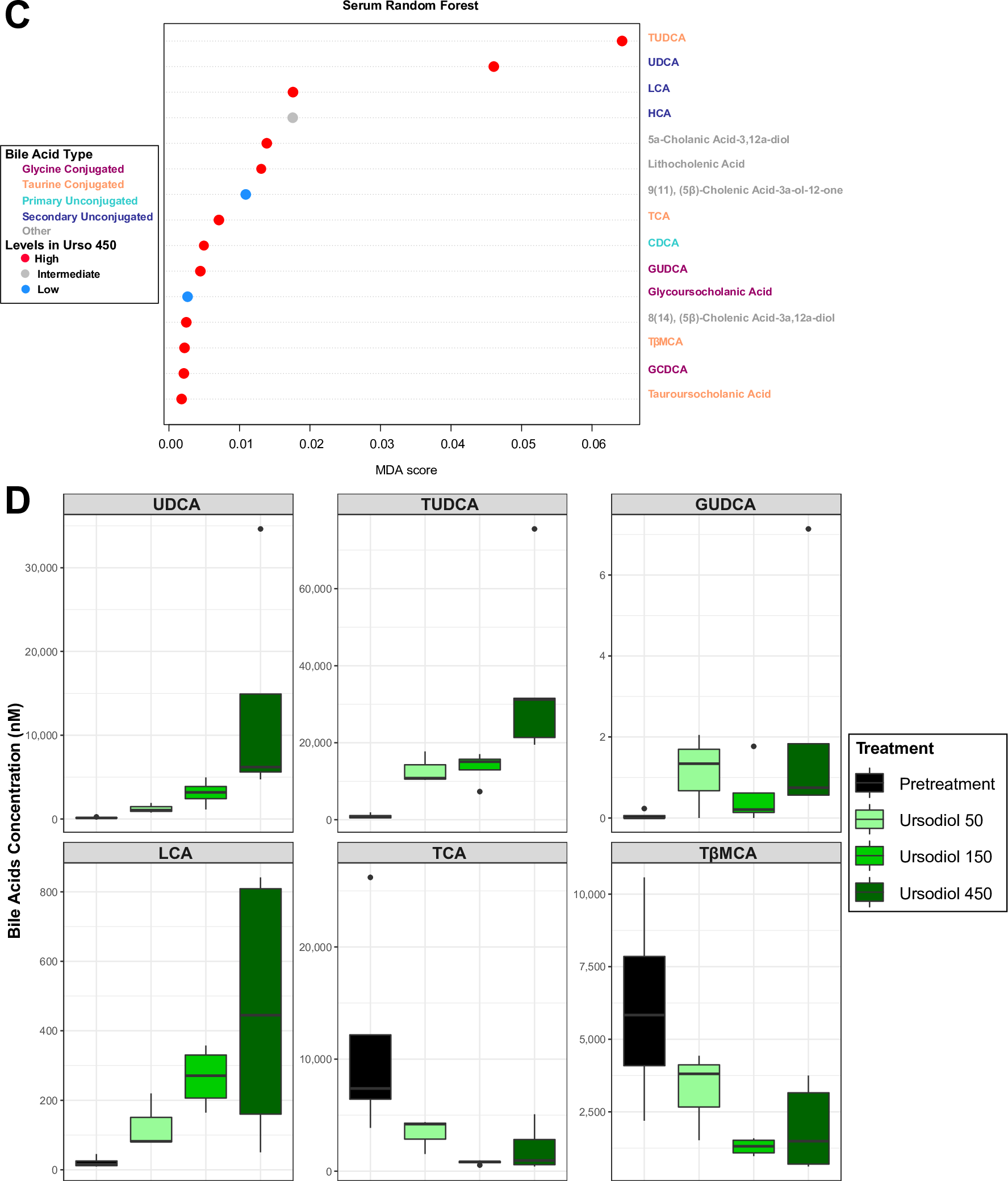

Supplement: S4 Fig — (A) NMDS ordination illustrates dissimilarity indices via Horn distances between bile acid profiles of serum samples. (B) Targeted bile acid metabolomics of murine serum was performed by UPLC-MS/MS and identified 38 distinct bile acids. (C) Variable-importance plot of the top 15 bile acids identified by Random Forest analysis. Bile acid points are color-coded for relative concentrations of each bile acid within the ursodiol 450 treatment group (red if their concentration is high in ursodiol 450 treatment, gray if they were intermediate, and light blue if the concentrations were low). Each bile acid name is colored coded based on bile acid type (purple indicates glycine conjugated, orange indicates taurine conjugated, teal indicates primary unconjugated, blue indicates secondary unconjugated, and gray indicates other type of bile acid). (D) Box and whisker plots of bile acids that were significantly altered in ursodiol treated mice compared to pretreatment in any of the sample types evaluated (based on a Two-way ANOVA with Dunnett’s multiple comparisons post hoc test). Data represents two independent experiments (pretreatment, n = 4; ursodiol 50, n = 3; ursodiol 150, n = 4; ursodiol 450, n = 6). (ZIP) [file pone.0246161.s004.zip › S4_Fig(2).tif]
